# Evaluation of Solid Particle Number Sensors for Periodic Technical Inspection of Passenger Cars

**DOI:** 10.3390/s21248325

**Published:** 2021-12-13

**Authors:** Anastasios Melas, Tommaso Selleri, Ricardo Suarez-Bertoa, Barouch Giechaskiel

**Affiliations:** European Commission, Joint Research Centre (JRC), 21027 Ispra, Italy; Anastasios.Melas@ec.europa.eu (A.M.); Tommaso.Selleri@ec.europa.eu (T.S.); Ricardo.Suarez-Bertoa@ec.europa.eu (R.S.-B.)

**Keywords:** periodical technical inspection, in-use vehicle emissions, particle number, diffusion charger, condensation particle counter, sub-23 nm particles

## Abstract

Following the increase in stringency of the European regulation limits for laboratory and real world automotive emissions, one of the main transport related aspects to improve the air quality is the mass scale in-use vehicle testing. Solid particle number (SPN) emissions have been drastically reduced with the use of diesel and gasoline particulate filters which, however, may get damaged or even been tampered. The feasibility of on-board monitoring and remote sensing as well as of the current periodical technical inspection (PTI) for detecting malfunctioning or tampered particulate filters is under discussion. A promising methodology for detecting high emitters is SPN testing at low idling during PTI. Several European countries plan to introduce this method for diesel vehicles and the European Commission (EC) will provide some guidelines. For this scope an experimental campaign was organized by the Joint Research Centre (JRC) of the EC with the participation of different instrument manufacturers. Idle SPN concentrations of vehicles without or with a malfunctioning particulate filter were measured. The presence of particles under the current cut-off size of 23 nm as well as of volatile particles during idling are presented. Moreover, the extreme case of a well performing vehicle tested after a filter regeneration is studied. In most of the cases the different sensors used were in good agreement, the high sub-23 nm particles existence being the most challenging case due to the differences in the sensors’ efficiency below the cut-off size.

## 1. Introduction

Strong scientific evidence on adverse health effects of particulate matter (PM) [1] has driven regulators to implement stricter limits to vehicles equipped with combustion engines because they were considered an important contributor of PM. In the European Union (EU), additionally to PM mass, a solid particle number (SPN) limit for particles >23 nm (SPN_23_) is also imposed to vehicles equipped with diesel and gasoline direct injection engines [2,3]. The SPN_23_ limit drove to the implementation of very efficient particulate filters. For example, diesel particulate filters have typically >99% solid particle number reduction efficiency [4], and gasoline particulate filters can also exceed >90% efficiency [5,6]. The stricter PM regulations in combination with the efficient PM exhaust after-treatment systems have resulted in reduced urban PM levels over the last years in European cities [7,8].

One issue that still remains open is the durability of exhaust after-treatment and tampering. Particulate filters become more efficient after their usage due to the formation of a soot cake and ash accumulation on their surface that traps soot particles [9]. Indeed, after the regeneration of a filter, the efficiency drops down significantly and increases de novo after the accumulation of soot [10]. However, defects of the particulate filters may reduce their trapping efficiency [11]. For example, laboratory and commercial fleet DPF (diesel particulate filter) failure studies have shown that uncontrolled filter regeneration with high temperature peaks in combination with the presence of ash may provoke thermal damages at the DPF’s substrate and more specifically pinholes, melts, and cracks [12,13,14]. Additionally, DPF tampering by vehicle owners has been reported with the aim of reducing fuel consumption and the need to perform the periodic regeneration [15]. Although these cases are a small percentage of the fleet, they can contribute significantly to the total fleet emissions. For example, a study of 300 diesel Euro 5 and Euro 6 vehicles sampled from the Belgian commercial fleet showed that 15% of high SPN emitters may increase the fleet emissions by a factor of 30 [16]. Another study found that 10% of highest SPN emissions can be responsible for 85% of the fleet emissions [17]. The determination of high emitters effect on the national fleet emissions may depend on several factors that increase the uncertainty but considering the DPF and GPF (gasoline particulate filter) efficiency, it is undeniable that vehicles with malfunctioning or removed particulate filters dominate the SPN emissions.

At EU level, the conformity of the vehicles to the emission limits over their useful lifetime (currently 160,000 km) is checked via the in-service conformity (ISC) testing (up to 100,000 km or 5 years). ISC testing is done to well-maintained vehicles following the type approval procedures. While ISC is conducted by the vehicle manufacturers and the type approval authorities, market surveillance, which was recently introduced in the regulation, can be done by independent institutes at a wider range of test conditions. A few well maintained vehicles with up to 160,000 km on the odometer are also selected [18]. Market surveillance is a very useful tool in order to detect defeat devices as well as assess the durability of after-treatment exhaust. Due to the high cost of laboratory and on-road testing, these tests cannot be applied to a mass scale. Thus, tampered or badly maintained vehicles are not controlled.

For large scale fleet monitoring on-board monitoring (OBM), remote sensing and periodical technical inspection (PTI) are the most appropriate tools. The idea of OBM is similar to the on-board diagnostics (OBD) of the vehicle [19]. However, instead of only checking the malfunctions of the vehicle, sensors are used to monitor the actual emissions. This concept has been successfully applied to heavy-duty vehicles in China [20]. In Europe discussions are on-going for OBM introduction in the Euro 7 regulation, but at the moment there are no robust particle number sensors. Remote sensing is wide spread [21,22]. From the big amount of data, durability issues at vehicle model level can be identified. It is also possible to identify high emitters. The application to particle number though is very limited [23].

In the framework of PTI, an opacity measurement is implemented for controlling the particulate filter but as modern engine and filter technologies have become very efficient, there are concerns on the sensitivity of this method [16,24]. Over the last years an informal technical working group for new periodic technical inspection (NPTI) procedures has been formed, aiming to develop methodologies for detecting DPF and de-NO_x_ aftertreatment technologies malfunctions. A methodology that seems to be very efficient for detecting tampered of malfunctioning particulate filters is the SPN measurement at idling. Especially for diesel vehicles the idling SPN concentrations may correlate well with SPN emissions during regulatory tests. Two studies that correlated regulatory tests measured in the laboratory and during on-road tests with low idling emissions (mostly extracted by the same tests) found that diesel vehicles that complied with regulation limits (6 × 10^11^ #/km) had <1 × 10^5^ #/cm^3^ low idling concentrations [25,26]. In another study, idling concentration in the order of 2.5 × 10^5^ #/cm^3^ corresponded to >10^12^ #/km and was proposed as a possible limit for PTI [24]. Low idling diesel SPN concentrations >1 × 10^5^ #/cm^3^ are correlated to >6 × 10^11^ #/km that is the current regulatory limit [17]. Instead, for gasoline vehicles the correlation is more difficult to be done [25] because they mainly produce particles during fuel enrichments [27] and not necessarily at idle. Nevertheless, the uncertainty of the methodology and the instrumentation has also to be taken into account in the determination of a limit.

Switzerland was the first country to introduce a high idling test for particles >23 nm (SPN_23_) for non-road machineries. For light and heavy duty vehicles the Netherlands was the pioneer and a PTI procedure measuring solid particles >23 nm will be introduced on the 1 July 2022. Belgium will also introduce the SPN measurement in the PTI in July 2022 and Germany will follow in January 2023. Emission limits, applicable vehicles, and testing procedures have differences from one country to the other. For example, the Netherlands has a limit of 10^6^ #/cm^3^ for Euro 5 and 6 vehicles and a 15 s measurement, while Germany 2.5 × 10^5^ #/cm^3^ only for Euro 6 vehicles with three repeats of 30 s measurements. A recent proposal from the VERT (Verification of Emission Reduction Technologies) association suggested a lower limit of 5 × 10^4^ #/cm^3^ both for diesel and gasoline vehicles equipped with particulate filter [28]. VERT proposes the performance of three 15 s measurements. In parallel to national initiatives also the European Commission is preparing a harmonized procedure but each member state will have the possibility to introduce the procedure as an additional measure within their own national competence. An important aspect of introducing a PTI test is the characteristics of the sensors used to perform the measurement. The sensors must be robust enough for the garage environment, simple in operation for non-expert staff, and of low cost. This large-scale production of PTI sensors needs some compromise regarding technical specifications that fulfill the required preciseness for detecting faulty or removed particulate filters. The main specifications of PTI devices that count solid particles are their efficiency to remove volatile particles and their lower detection size (that should be around 23 nm). Their background level and their maximum concentration are important aspects as well.

The approaches to these requirements are numerous. The heart of the sensors is the particle detector, which is typically based on optical particle counting after condensation of an alcohol on the pre-existing particles or measurement of electrical current after diffusion charging of the pre-existing particles. Condensation particle counters (CPCs) have a heated section where aerosol particles are exposed to supersaturated vapors and a colder section where vapors condense on particles and grow them to sizes that are detectable with optical methods. Different working fluids can be used for this application, most typically butanol and isopropanol. CPCs usually operate near ambient temperatures but recently also high temperature CPCs have been developed [29], but without any commercial system available at the time of writing. Their counting efficiency is near to unity at large sizes, their cut-off size may be influenced by the nature of the particles [30]. Diffusion chargers (DCs) utilize electrical detection of particles. Particles are charged by a corona charger which is typically unipolar but also systems with bipolar charging have been developed. After the corona charger, an electro-precipitator removes all free ions and finally the particles’ current is measured either with an electrometer or with a faraday cage [31,32]. Diffusion chargers do not use working fluids and can operate also at higher temperatures [33]. Compared to CPCs, DCs can measure higher particle concentrations but have higher background levels. Finally, their counting efficiency does not reach a plateau region as the CPC at large sizes due to the dependency of the charging efficiency on particles’ size.

In order to remove volatile particles and measure only solids, three different technologies are typically used: (i) heated (or evaporation) tube [34], (ii) thermodesorption using a thermal denuder [35], (iii) catalytic stripper that oxidizes hydrocarbons and optionally also traps sulfur compounds [36,37,38]. A review on volatile removal technologies can be found in [39].

Some systems have a sampling line; some others dilute at the sampling point. While differences to these approaches have been discussed in the literature due to different agglomeration and thermophoretic losses [40], in the PTI testing, where the exhaust gas temperatures are low and the concentrations also low, the differences should be small. High dilution may also be important in avoiding volatile artifacts when measuring particles below 23 nm [41]. For PTI systems though the dilution depends more on the upper limit they are designed to reach. Diffusion chargers usually have a high upper concentration limit and can measure even without dilution.

In the context of introducing a harmonized PTI procedure in the EU, the Joint Research Centre (JRC) of the European Commission performed an experimental campaign using PTI sensors from different manufacturers. Testing aimed to study the specifications of the sensors used for PTI applications and the level of particle emissions at low idling of different vehicles. A reference instrument compliant to the technical requirements of the type approval regulation was used for comparison with the different sensors. Different cases were studied; vehicles without a filter or malfunctioning filter, low idling after a DPF regeneration. Finally, sub-23 nm and volatile particles were measured in order to study their effect on the SPN sensors performance. The paper is divided in (i) the experimental section where we present the specifications of the sensors, the procedures, and the vehicles; (ii) the results were we focus on the performance of the sensors; (iii) the discussion section where we identify all parameters that are important for future regulation; and (iv) the conclusions.

## 2. Materials and Methods

### 2.1. Experimental Setup and Procedure

**Figure 1:**
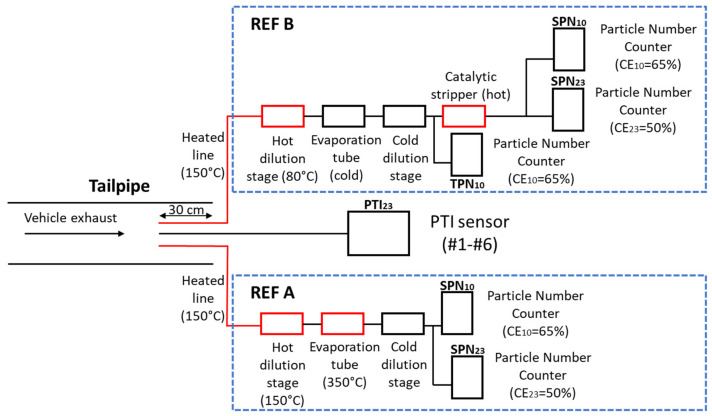
Schematic of the experimental setup. In red we show heated parts. Either ‘REF A’ or ‘REF B’ setups were employed while SPN-PTI sensors #1 to #6 were measuring sequentially.

Tests were performed in the vehicle emissions laboratory of the Joint Research Centre (JRC). The tested vehicles were placed in a laboratory with temperature varying from 20 to 27 °C. Figure 1 presents the experimental setup. All tests were performed at low idling with sampling directly from a depth of 30 cm in the tailpipe. Each of the six PTI sensors was measuring in parallel with reference systems.

The reference system (Nanomet 1, Testo, Titisee-Neustadt, Germany) was based on the technical requirements of the type approval EU regulation (2017/1151) and the Particle Measurement Programme (PMP) recommendations [42]. For this reason, quite often it is called the PMP system. It consisted of a 1 m hot sampling line at 150 °C, a hot dilution stage at 150 °C (dilution around 50:1), an evaporation tube operating at 350 °C, a secondary dilution stage (dilution around 5:1) at ambient temperature and a TSI (Shoreview, MN, USA) model 3790 Condensation Particle Counter (CPC) with 50% counting efficiency at 23 nm, CE_23_ = 50%. Additionally, a TSI model 3792 CPC with 65% efficiency at 10 nm, CE_10_ = 65%, was employed in parallel in order to measure solid particle number down to 10 nm. This reference setup will be called from now on ‘REF A’ and the SPN measurements with the 23 nm and 10 nm CPCs as SPN_23_ and SPN_10_, respectively. The ratio (SPN_10_ − SPN_23_)/SPN_23_ will be called sub-23 nm fraction.

The SPN concentrations were calculated with the Particle Concentration Reduction Factor (PCRF) that included the dilution factor and the average particle losses of particles with size 100 nm, 50 nm, 30 nm. The particle losses at 50 nm were 5% more than at 100 nm (PCRF_50_/PCRF_100_ = 1.05) and at 30 nm 29% more (PCRF_30_/PCRF_100_ = 1.29). The average PCRF was 300 in all tests. The sampling line did not add significant particle losses; for inlet flow 1.5 lpm and sampling line with length 1 m the diffusion losses of particles with size 23 nm is ~2%. The reference CPCs have been used in inter-laboratory exercises and during linearity checks for concentrations up to 10^4^ #/cm^3^ they had a slope between 0.9 and 1.1 and R^2^ > 0.99 [43,44]. For CPC concentrations >10^4^ #/cm^3^, (i.e., measured concentrations >3 × 10^6^ #/cm^3^) the accuracy of the reference system was not in the ±10% and the measurement uncertainty increased. These cases are indicated in the “Results” section. For SPN_10_ no additional correction was performed for the diffusion losses of sub-23 nm particles, as prescribed in the Global Technical Regulation (GTR) 15. Particle losses at 15 nm were higher than the average PCRF by a factor of 2.2.

For some tests, the reference system setup was modified: while the hot sampling line was kept at 150 °C, the primary hot dilution was set to 80 °C, and the evaporation tube was switched off. Downstream of the system, a TSI model 3792E CPC with 65% efficiency at 10 nm was measuring total particle number (both solid and semi-volatile) emissions (TPN_10_). With these sampling conditions nucleation of volatiles was probably suppressed, but nucleation of semi-volatiles was possible. A portion of the diluted aerosol flow was driven to a catalytic stripper model CS015 from Catalytic instruments (Rosenheim, Germany) with wall temperature 375 °C and then to the 23 nm CPC 3790 and 10 nm CPC 3792 in order to measure SPN_23_ and SPN_10_, respectively. Henceforth, this setup is called ‘REF B’ while the ratio (TPN_10_ − SPN_10_)/SPN_10_ is called volatile fraction. The PCRF of this setup was 225 (due to different temperatures used). The SPN_23_ and SPN_10_ measured downstream of the catalytic stripper and were additionally corrected by a factor of 1.4 to take into account the catalytic stripper’s particle losses at sizes 30 nm, 50 nm, and 100 nm.

The systems in setup ‘REF A’ or ‘REF B’, whichever was applicable, were measuring and logging continuously the SPN_23_ and SPN_10_ (and TPN_10_ with ‘REF B’) concentrations during low idling. The sampling point of both ‘REF A’ and ‘REF B’ was, similarly to the PTI sensors, 30 cm inside the tailpipe. After the ignition of the vehicle’s engine the PTI sensors were measuring sequentially for a predetermined time period that lasted from 15 (Sensors #1 to #4) to 45 (Sensor #5) seconds (3 repetitions of 15 s) according to the recommendations of the country of homologation that they followed. Sensors #1 to #4 had also a stabilization time of 15 s before measuring. Sensor #6 had only a continuous measurement option but its measurement time period was chosen to be similar to the rest of sensors and between 15 and 45 s. Before each measurement Sensors #1 to #4 performed an automatic or semi-automatic zero offset and leakage test. For Sensors #5 and #6 and the CPCs used at ‘REF A’ or ‘REF B’, whichever was applicable, the zero offset was checked before testing with a HEPA filter.

The measurement order of the PTI sensors changed from one test to another in order to have different concentration levels for all sensors. The duration of each idling test lasted from 10 to 30 min. Measurements were performed also during the cold start of the vehicles in order to have a wider range of concentrations. In some cases, the vehicle was switched off and on several times. The PTI sensors were compared against ‘REF A’ or ‘REF B’. Due to the absence of any possibility for post-process alignment, the data alignment was done with a timer during the test. Experimental time started when ‘REF A’ or ‘REF B’ logging started and for each PTI sensor we recorded the time of measurement. No on-board diagnostics (OBD) measurements were available during the testing campaign.

### 2.2. PTI Sensors

Sensors #1 to #5 were provided from the manufacturers to JRC for the testing campaign. The companies in alphabetical order were: Capelec (Montpelier, France) and Pegasor (Tampere, Finland), DEKATI (Kangasala, Finland), Mahle (Stuttgart, Germany), TEN (Baambrugge, The Netherlands), and TSI (Aachen, Germany). Some of them were commercially available while others prototypes. Sensor #6 was owned by JRC and it was the NPET of TSI, homologated for PTI measurements of non-road mobile machinery in Switzerland.

#### 2.2.1. Sampling and Measurement Technologies

Table 1 presents the PTI devices that were tested. For each device we report whether there was a heated sampling line or not, the dilution ratio (if applicable), the technology for removal of volatiles, the principle of particle detection, and finally the regulations each sensor complied with. Some of the sensors may comply also with other regulations (e.g., DE, BE) but we only report the country in which they applied for or obtained homologation at the time that this paper was written. Sensors #3 and #5 were prototypes and no specific country of homologation was defined.

Sensors #1 to #4 had a heated line at different temperatures in the range of 60 °C to 90 °C. Sensors #5 and #6 diluted the aerosol at the sampling point with a bifurcated flow diluter that filters part of the inlet flow and uses it as dilution air. Sensors #1 and #3 had no dilution while Sensor #2 had very low dilution. Sensor #4 diluted the aerosol flow 200:1 by using two ejector diluters with dilution ratio ~14. In order to remove volatile particles Sensor #1 included a thermal denuder, Sensors #2, #3, #4 included a heated or evaporation tube, and Sensors #5, #6 a catalytic stripper. Sensors #1 to #3 used a diffusion charger (DC) and #4 to #6 a condensation particle counter (CPC) as particle detectors.

All Sensors except from Sensor #3 were equipped with a water trap downstream the sampling line at the inlet of the device. All DC-based sensors used a unipolar charger to charge the particles. Sensor #2 sampled the aerosol using the Venturi effect while particles were charged after being mixed with a particle-free flow of positive ions generated by a corona charger. An ion trap collected ions that did not attach on particles while the particle number concentration was calculated by the escaping current which was continuously measured [45]. Sensor #3 used a diffusion charger, an ion trap, a diffusion particle collector, and an electrical detector while it operated at low pressure. Sensor #4 inlet flow was 1.2 lpm. It used a mixing type CPC [46] with cut-off size at 10 nm while particles in the range 10–23 nm were removed with a diffusion screen placed upstream the CPC. Sensors #5 and #6 had an inlet flow of 0.7 lpm but only 0.1 lpm was driven to the CPC. The rest of the inlet flow bypassed the CPC. The CPCs of the PTI sensors operated with isopropanol. For Sensors #4 and #6 the operator had to fill the working fluid while Sensor #5 incorporated a bag filled with the working fluid that had to be filled after certain number of measurements.

#### 2.2.2. Calibration Values

Table 2 summarizes the Swiss and Dutch technical requirements regarding the counting efficiency of the sensor at different particle sizes, the linearity of the sensor at a specific particle size, and finally the efficiency of removing tetracontane (C40) particles which are considered to represent (semi)volatile particles. VERT proposes the same specifications as Dutch regulation but additionally requires an additional efficiency of <2 at 200 nm. The German regulation will set the same specifications as those required in the current European regulation for SPN-PEMS (Regulation 2017/1154).

Table 2 presents also the counting efficiency, linearity, and volatile particle removal efficiency of the PTI sensors of the specific system that was used at the JRC campaign. The specification of the sensors changes according to the applicable regulation. The reported values were provided by the PTI sensors manufacturers and not tested by us.

### 2.3. Vehicles

In this study, six vehicles were tested at low idling. Table 3 presents for each vehicle, the model year and the Euro emissions standard it fulfilled, the existence of a particulate filter, the mileage, the engine displacement/power, and the fuel that was used. The notation we use is ‘V’ and the number of the vehicle. All vehicles were light duty homologated as M1 (=passenger cars) from Euro 3 to Euro 6d regulations. Five diesel and one gasoline with direct injection engine were tested. Note that for V4 an engine out flow was extracted and driven to the tailpipe where it was mixed with the DPF-out flow in order to simulate a malfunctioning DPF.

## 3. Results

### 3.1. Vehicles without Particulate Filter

**Figure 2:**
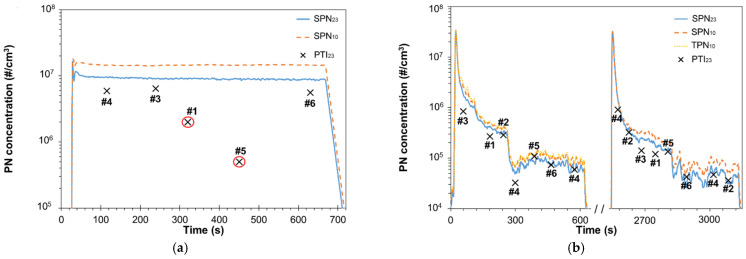
Particle number concentrations during low idling with cold start engine of direct injection vehicles without particulate filter: (**a**) diesel V6; (**b**) gasoline V5. Points in red circle show that the PTI sensors reported a threshold concentration that corresponds in automatic failure (2 × 10^6^ #/cm^3^ for NL or 5 × 10^5^ #/cm^3^ for DE).

Figure 2 presents the low idling emissions of two vehicles without particulate filter; a diesel (V6) and a gasoline direct injection (V5). Figure 2a plots the SPN_23_ and SPN_10_ emissions of V6 measured with the setup ‘REF A’. Both SPN_23_ and SPN_10_ of V6 low idling emissions were ~10^7^ #/cm^3^. This is in agreement with previous studies that reported >10^7^ #/cm^3^ [47,48] for diesel vehicles without DPF. Note that the reference system measured >3 × 10^6^ #/cm^3^ so the SPN_23_ was probably underestimated. Some PTI devices reported SPN emissions that were higher than the limit proposed by the (corresponding) regulation they follow. Specifically, Sensors #1 and #2 report up to 2 × 10^6^ #/cm^3^ twice as high the Dutch limit, and the Sensor #5 up to 5 × 10^5^ #/cm^3^. This is not the upper concentration limit of the sensors but the threshold value they use to report fail of the vehicle. In the cases that PTI sensors reached this upper limit we added a red circle (see Figure 1a). Even if PTI sensors underestimated in some cases the SPN_23_, they all reported that the vehicle failed to comply with regulation due to SPN concentrations higher than 2 × 10^6^ #/cm^3^ (Sensor #5 has a limit at 5 × 10^5^ #/cm^3^). Thus, all sensors detected that this vehicle was a high emitter. Sensor #2 was not available during this test.

Figure 2b reports the emissions of V5 after two engine ignitions; one with cold engine and one with hot that was performed approximately 30 min after the vehicle was switched off. During the first test the setup ‘REF B’ was used and SPN_23_, SPN_10_ and TPN_10_ were measured, while during the second test only SPN_23_ and SPN_10_ were measured with ‘REF A’. SPN_23_ emissions were initially >>10^6^ #/cm^3^ while after the first 300 s they were ~3 × 10^5^ #/cm^3^. The 10 nm to 23 nm concentration (SPN_10–23_) was ~35% more than SPN_23_ throughout the test (see also Section 3.6). After 600 s of idling, the concentration decreased to 6.5 × 10^4^ #/cm^3^. A very similar concentrations profile was also observed during the second test, the lowest SPN_23_ being 3.5 × 10^4^ #/cm^3^. Interestingly, the concentration was not stable throughout the test but fluctuated significantly. 

In general, all sensors were precise enough. The difference of Sensors #2, #5, and #6 to SPN_23_ were within ±19% and of Sensor #1 within ±34%. The highest differences compared to SPN_23_ were 54% and 38% for instruments #3 and #4, respectively. The variability of the emissions (defined as standard deviation of SPN_23_ divided by average SPN_23_ for the specific time period) was 15% and 6% for these two tests, respectively. The measurement with the device #4 was repeated at a more stable idling emissions point and the deviation with ‘REF B’ system decreased to 7%. During the second test, the highest PTI sensors deviation occurred for Sensor #4 but the SPN_23_ standard deviation was >40%. Even after 400 s the SPN_23_ standard deviation was >10% that resulted in high uncertainty for the PTI sensors results that were measuring only for a frame of 15 s (except for Sensor #5 and Sensor #6).

### 3.2. Malfunctioning DPF

**Figure 3:**
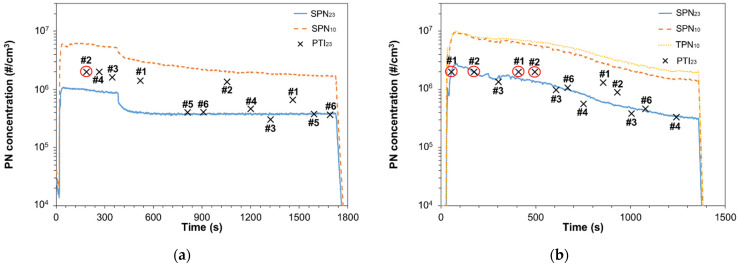
Particle number concentration during low idling with cold start engine for: (**a**) Euro 4 diesel V3; (**b**) Euro 6d with DPF bypass V4. Points in red circle show that the PTI sensors reported a threshold concentration that corresponds in automatic failure (2 × 10^6^ #/cm^3^ for NL or 5 × 10^5^ #/cm^3^ for DE).

Figure 3 plots the emissions of two vehicles with reduced DPF efficiency. Figure 3a presents the SPN_23_ and SPN_10_ emissions of V3 (setup ‘REF A’) which was a Euro 4 with mileage >200,000 km. Initially, SPN_23_ emissions were higher than 10^6^ #/cm^3^ and gradually decreased to 8.5 × 10^5^ #/cm^3^. After 380 s, SPN_23_ decreased steeply to ~3.8 × 10^5^ #/cm^3^ and then stabilized. When SPN emissions stabilized, SPN_10–23_ was ~350% more than SPN_23_ for V3 (see Section 3.6). Initially, SPN_10_ and TPN_10_ concentrations were >3 × 10^6^ #/cm^3^ but after the first 380 s they decreased to their accurate measurement range. The PTI sensors had a good agreement with the SPN_23_ measurements when emissions stabilized except for Sensor #2 and to a lesser degree for Sensor #1 which overestimated the concentrations. In one case, a PTI sensor indicated that emissions were higher than 2 × 10^6^ #/cm^3^ that results an immediate failure in the NL regulation. For this case we added a red circle in Figure 3a.

Figure 3b plots the SPN_23_, SPN_10_ and TPN_10_ emissions of V4 (setup ‘REF B’). For this vehicle an engine-out flow was bypassed and mixed with the flow downstream of the DPF. The concentration was initially 2.8 × 10^6^ #/cm^3^ and gradually decreased down to 2.7 × 10^5^ #/cm^3^. The SPN_23_ concentration range spanned over the limits imposed by both Netherlands (1.0 × 10^6^ #/cm^3^) and Germany (2.5 × 10^5^ #/cm^3^). Engine- and DPF-out SPN_23_ emissions of V4 were found to be ~5 × 10^6^ #/cm^3^ and ~5 × 10^3^ #/cm^3^, respectively. Thus, the filter bypass applied in our study reduced the DPF efficiency from ~99.9% to ~93%. Similar to Figure 3a, when PTI sensors reported the failure value of 2 × 10^6^ #/cm^3^ we added a red circle. When concentrations were near the Dutch limit, the PTI sensors were accurate detecting those cases where the emissions exceeded the limit. Specifically, Sensors #1, #2, #3, #4, and #6 detected all the cases that SPN_23_ was higher than the Dutch limit (1.0 × 10^6^ #/cm^3^). Sensor #5 had a limit of 5 × 10^5^ #/cm^3^. When SPN_23_ decreased to values lower than 1.0 × 10^6^ #/cm^3^_,_ the sensors were still in good accuracy but in two cases Sensors #1 and #2 overestimated SPN_23_ by 119% and 68%, respectively. Sensor #5 did not measure in the presented test. Its efficiency when measuring the PN emissions of V4 will be discussed in Section 3.6.. Note that SPN_10_ and TPN_10_ measurements during the first ~600 s had high uncertainties due to elevated concentration values (>3 × 10^6^ #/cm^3^).

### 3.3. After DPF Regeneration

**Figure 4:**
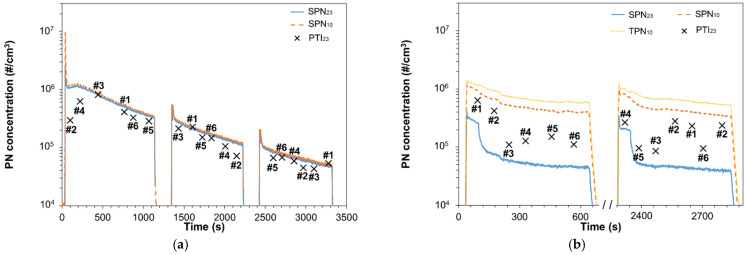
Particle number concentration during low idling: (**a**) After regeneration: V2 cold start and two hot starts. Hot engine ignitions were done few minutes after switching off the engine; (**b**) High sub-23 nm fraction: V1 cold start and one hot start.

Figure 4a plots the SPN_23_ and SPN_10_ low idle concentrations of diesel vehicle V2 (‘REF A’) and compares them to PTI sensors measurements. After the first cold start engine ignition, two more ignitions at hot engine conditions followed (around 1300 s and 2400 s, respectively). The engine remained switched off only for few minutes before the two hot engine ignitions. The concentration was initially very high, >10^6^ #/cm^3^, and gradually decreased two orders of magnitude. The profile indicates that the DPF efficiency was increasing during the test and thus, our measurements were performed right after a regeneration. No OBD was available to confirm our assumption.

Sensors #1 and #3 were very precise while the rest of the sensors underestimated the SPN_23_ emissions. During the first seconds of the first ignition, Sensors #2 and #4 underestimated SPN_23_ significantly but their deviation decreased at the second and third tests at levels near the accuracy requirement of NL regulation (~25%).

### 3.4. High Sub-23 nm Fraction

Figure 4b plots SPN_23_, SPN_10_, and TPN_10_ emissions of V1 (setup ‘REF A’) and compares them to the PTI sensors measurements. Initially the SPN_23_ concentration was >2 × 10^5^ #/cm^3^. After ~80 s the SPN_23_ decreased steeply to ~6 × 10^4^ #/cm^3^ and then gradually down to 4 × 10^4^ #/cm^3^. The concentration of particles below 23 nm were almost seven times the concentration of particles >23 nm. This means that the mean particles size was below 23 nm. All PTI sensors overestimated significantly the SPN_23_ emissions. A second measurement was performed with hot engine ~25 min after switching off the engine. The results were similar to the first measurement. The sensor with the smallest deviation was Sensor #3 (85–91% difference), while the rest deviated 222–433% (Sensor #1), 464–515% (Sensor #2), 28–141% (Sensor #4), 83–226% (Sensor #5), and 128–144% (Sensor #6).

### 3.5. Total Particles

TPN_10_ concentrations (solid and volatile particles) were measured with setup “REF B” for V1, V4, and V5. Table 4 summarizes the volatiles fraction calculated as the absolute value of the ratio of SPN_10_-TPN_10_ to SPN_10_. Volatiles fraction is very low for the gasoline V5 (6%), while for the diesel vehicles V1 and V4 are 46% and 56%, respectively. The volatile fraction at low idling was low for these three vehicles. The highest fraction was detected for V4 that had also an engine-out flow that did not pass through the diesel oxidation catalyst (DOC) that oxidizes hydrocarbons.

### 3.6. Summary of Results

Table 4 summarizes the SPN_23_, SPN_10_, TPN_10_, the sub-23 nm fraction of solid particles emitted by the tested vehicles defined as SPN_10–23_/SPN_23_, and the volatile fraction. The stabilized parts of the concentrations were used to calculate the fractions. The standard deviation of the calculated fractions was <3% except for V5 where standard deviation was 7%.

The SPN_23_ levels were from 4.0 × 10^4^ #/cm^3^ (V1) up to 8.9 × 10^6^ #/cm^3^ for the vehicle without DPF (V6). The vehicle with a fraction of the exhaust bypassing the DPF had a concentration of 2.7 × 10^5^ #/cm^3^ (V4) and the high mileage DPF vehicle a slightly higher (V3). Both of them were higher than Germany’s limit (2.5 × 10^5^ #/cm^3^), but lower than the Dutch limit (1.0 × 10^6^ #/cm^3^).

The sub-23 nm fraction was very high for V1, V3, and V4 (>150%). In these cases, the inclusion of sub-23 nm particles in the regulation may change the status of a vehicle from ‘pass’ to ‘fail’. For example, V1 had SPN_23_ 4.0 × 10^4^ #/cm^3^, but SPN_10_ 3.5 × 10^5^ #/cm^3^, which is higher than the Germany’s limit of 2.5 × 10^5^ #/cm^3^. V4′s SPN_23_ was at the limit, but SPN_10_ exceeded the limit by far. Such high sub-23 nm fractions indicate the importance of the cut-off size of the PTI sensors. This was clear with the tests of V1 that had the higher sub-23 nm fraction (Figure 4b).

**Figure 5:**
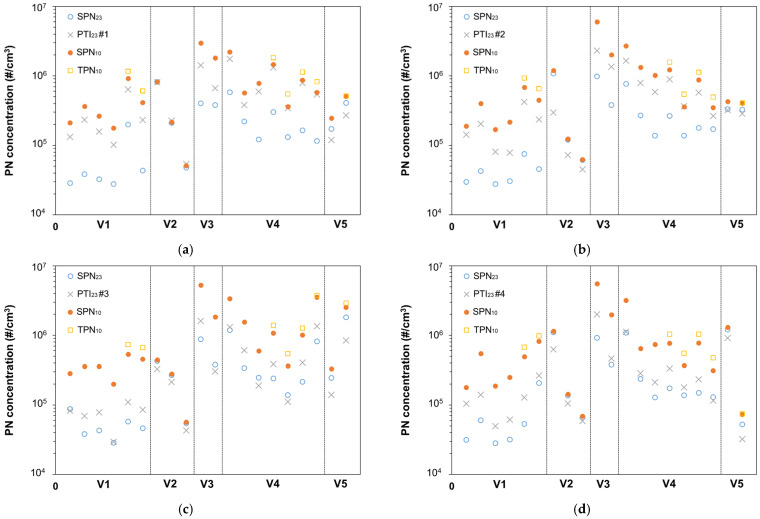

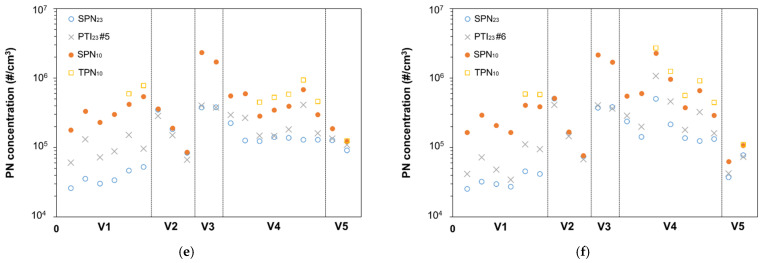
Summary of PTI sensors measurements and comparison with SPN_23_ measured with a PMP compliant system. SPN_10_ and TPN_10_ (when available) are also provided. (**a**) PTI #1; (**b**) PTI #2; (**c**) PTI #3; (**d**) PTI #4; (**e**) PTI #5; (**f**) PTI #6.

Figure 5 correlates the PTI sensors to SPN_23_ measurements. Additionally, we plot the SPN_10_ and, when available, the TPN_10_ in order to study the effect of sub-23 nm and volatile particles on the performance of PTI sensors. The vertical solid lines divide the number concentrations for different vehicles. V6 is not plotted because most sensors saturated.

**Figure 6:**
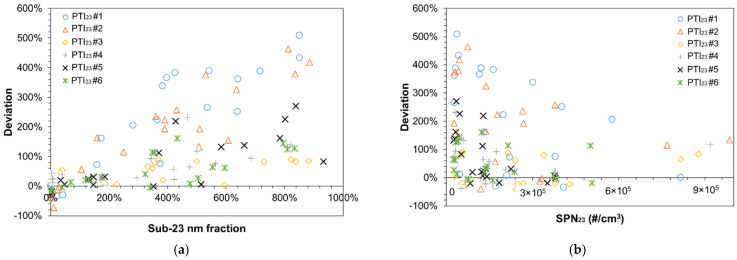
Summary of the deviation of the PTI sensors plotted against: (**a**) the sub-23 nm fraction (SPN_10_ − SPN_23_)/SPN_23_); (**b**) the solid particle number of particles down to 23 nm (SPN_23_).

Figure 6a plots the deviation of the PTI sensors compared to the SPN_23_ concentrations in function of the sub-23 nm fraction. When the sub-23 nm fraction is <100% the accuracy of the PTI sensors is very good. When the sub-23 nm fraction is 200% or more the deviations become bigger, especially for Sensors #1 and #2. CPC based sensors and DC-based Sensor #3 were less influenced by the presence of small particles. Figure 6b plots the deviation of the PTI sensors against the SPN_23_ measured with the reference system. Measurement uncertainty is in general higher at lower concentrations but there is no clear trend between the measured concentration and the sensors’ deviation. The scatter is due to the sensitivity of the sensors to the sub-23 nm fraction as described in Figure 6a.

## 4. Discussion

This is one of the first studies that assessed sensors for the PTI of vehicles. Previous studies used prototypes [48,49,50], or the Swiss approved sensor [24]. Here from the six sensors tested, one had the approval (certificate) for the Swiss PTI (Sensor #6), two were prototypes without a specified country for homologation (Sensors #3 and #5), and three sensors (Sensors #1, #2 and #4) had either approval (certificate) for PTI testing or were ready for approval in Netherlands. All of the sensors fulfilled the technical requirements of the countries they had the approval from, or the VERT recommendations. In principle, depending on the regulation, an uncertainty of ±30% is expected from the technical specifications (see Table 2). Even though such small differences were indeed seen, there were some cases of much higher differences (up to 5 times higher). The key message of this study was that the reason of these high differences was the high (or low) sensitivity of the sensors to particles smaller than 23 nm (Figure 6a), which is the current lower size in the regulations. Most importantly, these sub-23 nm particles were “solid” and not volatiles. The implications of this finding will be discussed in more details below.

### 4.1. The Role of Sub-23 nm Particles in the PTI Sensors Deviation

Up to 160% sub-23 nm fraction could be handled acceptably by the PTI sensors (Figure 6a). At 200% sub-23 nm value, two DC−based sensors started deviating by >150%. The CPC-based systems had high deviations (>100%) at >400% sub-23 nm values. The declared counting efficiencies of the PTI sensors at 23 nm presented in Table 2 do not justify these high differences. Thus, the rationale for the differences observed is possibly due to the efficiency of the sensors at sizes below 23 nm. The counting efficiency of CPCs at sizes below the cut-off size decreases steeper than diffusion chargers. A recent study on the uncertainty of regulatory particle number measurements [40] found that at 50 nm a PMP system (CPC-based) and a portable emissions measurement system, SPN-PEMS (both CPC- and DC-based), have very similar efficiencies (~90%). Instead, at 15 nm the PMP system would typically measure in the range 16–23% while CPC-based PEMS ~24% and DC-based ~33%. Thus, a DC-based PEMS may measure even double sub-23 nm particles concentration compared to a high losses PMP system with 16% efficiency at 15 nm. Even if PTI sensors are not necessarily equal to PEMS systems the aforementioned differences give important input on the differences observed in this study. A previous study [47] has calculated the possible under- and over-estimation of SPN_23_ of PTI instruments as a factor of the geometric mean diameter. The upper maximal SPN_23_ measurement deviation for geometric mean diameters in the range 35–77 nm was estimated to be 18% to 84%; higher geometric mean diameters and lower geometric standard deviations resulted higher deviations. In addition to the sub-23 nm effect on the sensors’ accuracy we also studied possible linearity issues (Figure 6b) but no clear trend was observed between deviation and concentration. Moreover, it was not clear whether the sensors that overestimated the emissions were affected by volatile particles. The total particles were measured for the two diesel vehicles with high sub-23 nm fraction. They were approximately 50% higher than the solid particles. As the concentration of solid particles below 23 nm was very high, we believe that any volatile particles would be mainly condensed on the existing solid nanoparticles, rather than forming a separate volatile nucleation mode. During idling of diesel engines, the air to fuel ratio is very high and small volatile particles fraction is emitted compared to other engine operation conditions [51]. More studies are needed to assess the volatile removal efficiency of the sensors under realistic and extreme conditions (i.e., with existence of nucleation mode particles).

### 4.2. Sub-23 nm Particles at Idling

The second point that needs to be discussed is what are the particles below 23 nm and whether their concentration is high. Diesel engines typically produce size distributions with geometric mean diameters in the range of 50–70 nm [42]. Thus, in general they have a low sub-23 nm particles fraction. Formation of sub-23 nm particles have been recently reported during urea or ammonia injection [52]. These particles were also found to carry high charge at high exhaust gas temperatures [53]. In our study, half of the diesel vehicles were not equipped with SCR (selective catalytic reduction for NO_x_). For the tests with the vehicles with SCR (V2, V4), due to the low exhaust gas temperatures at idling, we believe that no urea injection took place and due to the low exhaust gas temperature no such particles were formed even if any ammonia desorbed from the catalyst. Indeed, V2 had very low sub-23 nm values while particle emissions of V4 mainly originated by the engine-out flow and, thus, they were not influenced by possible urea injection. Furthermore, we did not observe any significant variation of the sub-23 nm value that would indicate release of ammonia (Figure 3b). Another case with high concentration of solid particles below 23 nm is idling. This has been shown and confirmed repeatedly in the literature and it is assumed that they are heavy polyaromatic hydrocarbons (PAHs) that cannot evaporate at 350 °C [54]. The concentration of these particles was extremely high for vehicle V1 (7.5 times of SPN_23_), but still high for V3 and V4 (1.5 to 3.5 times of SPN_23_). If we also consider the particle losses of sub-23 nm particles due to diffusion (not corrected in this study), their fraction would be even higher (1.5–2 times). For the remaining diesel vehicles, the fraction of particles below 23 nm was <65%. What is important to note is that the high concentration of particles below 23 nm at idling does not extrapolate to other engine operation modes or the type approval cycle. Dedicated tests with V1 showed that the SPN_23_ type approval cycle emissions were 1.48 × 10^11^ #/km, but for the same cycle the SPN_10_ emissions were 1.85 × 10^11^ #/km. Thus, the approximately 700% higher SPN_10_ idle concentration corresponded to only 27% higher SPN_10_ cycle emissions. There was also no correlation between SPN_23_ idle concentrations and sub-23 nm fraction (see Table 4). Combining this lack of correlation with the lack of correlation of idle sub-23 nm fraction and type approval cycle sub-23 nm fraction, it can be concluded that the PTI sensors need to avoid counting this fraction.

### 4.3. The Importance of PTI Sensors Efficiency in the Sub-23 nm Size Region

The third question that needs to be answered is whether this sensors′ concentration uncertainty at low particle sizes is important. For vehicles having low idle emissions (i.e., <5 × 10^4^ #/cm^3^) an error on the order of 5 times (e.g., V1), will bring the result close to the German limit. For a vehicle close to the German limit an error on the order of 3 is still below the Dutch limit (e.g., V3 or V4). When discussing limits, the uncertainty of the whole procedure should also be taken into account. The idle concentration can give an estimation of the type approval cycle emissions (factor 10^7^ cm^3^/km), but this factor has an uncertainty margin of at least 2 (for diesel vehicles); for gasoline vehicles the factor is much higher [25]. Thus, a vehicle that is close to the type approval limit (6 × 10^11^ #/km), taking into account the factor 2 would have idle concentration of up to 1.2 × 10^5^ #/cm^3^. By setting a limit of 2.5 × 10^5^ #/cm^3^ (German regulation) an additional factor of 2 is permitted for the PTI sensors uncertainty. In our study two vehicles were close to the German limit: V3 (old DPF) and V4 (bypassed DPF). All PTI sensors correctly identified that idling emissions of these two vehicles were >2.5 × 10^5^ #/cm^3^, but in many cases the SPN_23_ emissions were significantly overestimated. V3 with idle concentration of 3.8×10^5^ #/cm^3^ was precisely assessed by Sensors #5 and #6 (within 2%). The average deviation of Sensors #3 and #4 was 32% and 70%, respectively. Sensors #1 and #2 overestimated >150% due to the high fraction of particles below 23 nm (346%). SPN_23_ of V4, when reducing the DPF efficiency from 99.9% to 93%, was 2.7 × 10^5^ #/cm^3^ and the sub-23 nm fraction was 158%. Similar to V1 all PTI sensors overestimated the SPN_23_; on average (for each sensor’s measurements) Sensors #3−#6 were within 52% while Sensors #1 and #2 overestimated by 273% and 187%, respectively.

One vehicle had higher emissions than the Dutch limit: V6 (no DPF). This vehicle had emissions close to 1 × 10^7^ #/cm^3^. Except from Sensor #5 that reported the failure threshold value in the German regulation (5 × 10^5^ #/cm^3^), all sensors detected that this vehicle had >2 × 10^6^ #/cm^3^ that results in an immediate fail in the Dutch regulation. The idle concentrations of <5 × 10^4^ #/cm^3^ of V2 were measured accurately by all sensors within 20% (sub-23 nm fraction 5%). On the other hand, the idle concentrations of V1, which were at the same levels (4 × 10^4^ #/cm^3^) were not determined accurately by all sensors due to the high sub-23 nm fraction (770%). Sensors #3 and #6 measured <1 × 10^5^ #/cm^3^, but Sensors #1, #2, #4 and #5 above; Sensors #1 and #2 even above the German limit in some cases. V1 type approval cycle emissions are well below the limit. Thus, with the current Dutch regulation technical requirements, the German limit might result in some false “fails”. Any limit at this or lower level needs more rigorous characterization of the cut-off curve of the sensors.

### 4.4. Gasoline Vehicles

The discussion focused on diesel vehicles, because the upcoming Dutch, German, and Belgian PTI regulations will apply only to diesel vehicles. The reason is that tampering or malfunction of DPFs will have a significant impact on the emissions, because the engine out emissions are very high (around 10^14^ #/km) [24]. On the other hand, the SPN_23_ emissions of gasoline vehicles even without any particulate filter are near the regulation limit (around 10^12^ #/km), while modern gasoline vehicles may emit one order of magnitude lower SPN_23_ [3,55]. Thus, the detection of existence or malfunctioning of the filter is very difficult. A previous study also showed that is difficult to find a good correlation between idle concentration and type approval emissions [25]. More studies in this direction are necessary.

## 5. Conclusions

In this study, the measurement of low idling emissions of different vehicles was performed with six SPN sensors designed for periodical technical inspection (PTI) applications and a reference system that measured >23 nm (SPN_23_) and >10 nm (SPN_10_), and in some cases also the total particle number >10 nm (TPN_10_). Our scope was twofold; to evaluate the efficiency of the PTI_23_ sensors in the context of the limits set by different current or future PTI regulations and to provide input on the procedures. The cases we studied were: high sub-23 nm particles and volatiles fraction, emissions after a DPF regeneration, and vehicles without particulate filter or with a malfunctioning filter.

SPN_23_ low idling emissions of a diesel vehicle without a DPF were around 1 × 10^7^ #/cm^3^, one order of magnitude higher than the Dutch limit, and easily detectable by all sensors. For malfunctioning DPFs we found emissions SPN_23_ slightly higher than the German limit of 2.5 × 10^5^ #/cm^3^. In one case (V4), the DPF efficiency of a well performing vehicle was controllable reduced from ~99.9% to ~93% and SPN_23_ emissions were 2.7 × 10^5^ #/cm^3^. SPN_23_ emissions were very high after a DPF regeneration (even > 3.8 × 10^5^ #/cm^3^) and gradually decreased to <1 × 10^5^ #/cm^3^ showing the necessity of a short conditioning (e.g., some minutes of driving) of the vehicle in these cases. Finally, the SPN_23_ low idling emissions of a GDI vehicle without a filter were much lower (<1 × 10^5^ #/cm^3^) than the currently proposed limits pointing the necessity of performing more studies on both the procedures and the PTI limit for gasoline vehicles.

Our results suggest that PTI requirements for PN measurements may be met by both CPC- and DC-based sensors. All sensors detected high emitters (>1 × 10^6^ #/cm^3^) and for low sub-23 nm fractions their accuracy was within 50% in most of the cases. The highest deviations of the PTI_23_ sensors were observed when the sub-23 nm fraction was high. The SPN_10–23_ was even 775% higher than SPN_23_ in one case (V1), much higher than typical values for diesel vehicles, showing that diesel engines may emit high concentrations of nonvolatile nucleation particles during idling. Two out of the three DC-based sensors (Sensors #1 and #2) were mostly affected by the presence of sub-23 nm particles and overestimated significantly SPN_23_ resulting in false ‘fails’ in case that a limit in the order of 2.5 × 10^5^ #/cm^3^ will be imposed. The volatile particles down to 10 nm were ~50% for two diesel vehicles and 6% for a gasoline (G-DI) vehicle more than SPN_10_. In these three cases no correlation was found between volatiles and sensors deviation.

## Figures and Tables

**Table 1:** Sampling and measurement technologies used at the PTI sensors.

PTI	Sampling Line	Dilution (Temp.)	Volatile Particle Remover	Particle Detector	Certification
#1	Heated (75 °C)	No	Thermal denuder (150 °C)	DC	NL
#2	Heated (90 °C)	Venturi (150 °C)	Evaporation tube (200 °C)	DC	NL
#3	Heated (60 °C)	No	Evaporation tube (300 °C)	DC	N/A
#4	Heated (70 °C)	200:1 (ambient)	Evaporation tube (250 °C)	CPC	NL
#5	Not heated	20:1 (ambient)	Catalytic stripper (350 °C)	CPC	N/A
#6	Not heated	10:1 (ambient)	Catalytic stripper (350 °C)	CPC	CH

CH = Switzerland; CPC = Condensation Particle Counter; DC = Diffusion Charger; N/A = not available; NL = Netherlands.

**Table 2:** Requirements for PTI sensors at different regulations (CH, NL, VERT) and calibration values of the PTI sensors as provided by the manufacturers.

	Counting Efficiency				Linearity (80 nm)	VRE
	23 nm	50 nm	80 nm	200 nm	Polydisperse	30 nm Tetracontane
CH	<0.50	*	0.70–1.30	<1.30		>90% (<10^5^ #/cm^3^)
NL	0.20–0.60	0.60–1.30	0.70–1.30	-	0.75–1.25	>95% (<10^5^ #/cm^3^)
VERT	0.20–0.60	0.60–1.30	0.70–1.30	<2.00	0.75–1.25	>95% (<10^5^ #/cm^3^)
#1	0.34	0.75	1.00	-	1.03 (80 nm)	>95% (10^4^ #/cm^3^)
#2	0.47	0.86	1.12	-	0.99 (76 nm)	>95% (10^5^ #/cm^3^)
#3	0.43	0.76	1.00	1.67	0.99 (37–56 nm)	100% (>10^4^ #/cm^3^)
#4	0.40	0.90	1.00	1.15	0.998 (poly)	99.9% (3.5 × 10^4^ #/cm^3^)
#5	0.55	0.95	1.02 (70 nm)	1.04	N/A	N/A
#6	0.33	0.55 (41 nm)	-	-	1.04 (no size info)	>99%

* >0.4 at 41 nm. VRE = Volatile Removal Efficiency. In brackets the concentration of volatile particles; N/A = not available.

**Table 3:** Main characteristics of tested vehicles.

Code	Euro	Fuel	Year	Mileage (km)	Engine Displacement (cm^3^)	Power (kW)	Particulate Filter
V1	6b	Diesel	2017	23,540	1.560	88	Yes
V2	6d	Diesel	2019	4.100	1.999	132	Yes
V3	4	Diesel	2009	209,000	1.997	100	Yes
V4	6d	Diesel	2020	4.200	1.968	110	Yes ^1^
V5	5b	Gasoline DI	2012	151,831	1.197	77	No
V6	3	Diesel	2004	286,000	2.993	150	No

^1^ An engine-out flow was available and mixed with the DPF-out flow. DI = Direct Injection. DPF = Diesel Particulate Filter.

**Table 4:** Mean concentrations and sub-23 nm and volatile fractions.

Vehicle	Comment	SPN_23_ (#/cm^3^)	SPN_10_ (#/cm^3^)	Sub-23 nm Fraction	TPN_10_ (#/cm^3^)	Volatiles Fraction
V1	DPF (high sub-23)	4.0 × 10^4^	3.5 × 10^5^	775%	4.5 × 10^5^	47%
V2	DPF (after regen.)	4.8 × 10^4^	5.0 × 10^4^	5%	-	
V3	DPF (old)	3.8 × 10^5^	1.7 × 10^6^	346%	-	
V4	DPF (bypass)	2.7 × 10^5^	7.0 × 10^5^	158%	1.1 × 10^6^	57%
V5	G-DI (no filter)	7.6 × 10^4^	1.0 × 10^5^	35%	1.1 × 10^5^	6%
V6	No DPF	8.9 × 10^6^	1.4 × 10^7^	63%	-	

Sub-23 nm fraction = (SPN_10_ − SPN_23_)/SPN_23._ Volatile fraction = (TPN_10_ − SPN_10_)/SPN_10._

## Data Availability

Data available upon request from the corresponding author.

## References

[1] Pope C.A., Burnett R.T., Thun J.M., Calle E.E., Krewski D., Ito K., Thurston G.D. (2002). Lung Cancer, Cardiopulmonary Mortality, and Long-Term Exposure to Fine Particulate Air Pollution. J. Am. Med. Assoc..

[2] Giechaskiel B., Maricq M., Ntziachristos L., Dardiotis C., Wang X., Axmann H., Bergmann A., Schindler W. (2014). Review of Motor Vehicle Particulate Emissions Sampling and Measurement: From Smoke and Filter Mass to Particle Number. J. Aerosol Sci..

[3] Giechaskiel B., Joshi A., Ntziachristos L., Dilara P. (2019). European Regulatory Framework and Particulate Matter Emissions of Gasoline Light-Duty Vehicles: A Review. Catalysts.

[4] Mayer A., Matter U., Scheidegger G., Czerwinski J., Wyser M., Kieser D., Weidhofer J. (1999). Particulate Traps for Retro-Fitting Construction Site Engines VERT: Final Measurement and Implementation. SAE Tech. Pap..

[5] Adam F., Olfert J., Wong K., Kunert S., Richter J.M. (2020). Effect of Engine-out Soot Emissions and the Frequency of Regeneration on Gasoline Particulate Filter Efficiency. SAE Tech. Pap..

[6] Boger T., Glasson T., Rose D., Ingram-Ogunwumi R., Wu H. (2021). Next Generation Gasoline Particulate Filters for Uncatalyzed Applications and Lowest Particulate Emissions. SAE Tech. Pap..

[7] Font A., Guiseppin L., Blangiardo M., Ghersi V., Fuller G.W. (2019). A Tale of Two Cities: Is Air Pollution Improving in Paris and London?. Environ. Pollut..

[8] Murzyn F., Sioutas C., Cavellin L.D., Joly F., Baudic A., Mehel A., Cuvelier P., Varea E., Rouland B.P. (2021). Assessment of Air Quality in Car Cabin in and around Paris from On-Board Measurements and Comparison with 2007 Data. J. Aerosol Sci..

[9] Konstandopoulos A.G., Kostoglou M., Skaperdas E., Papaioannou E., Zarvalis D., Kladopoulou E. (2000). Fundamental Studies of Diesel Particulate Filters: Transient Loading, Regeneration and Aging. SAE Tech. Pap..

[10] Giechaskiel B. (2020). Particle Number Emissions of a Diesel Vehicle during and between Regeneration Events. Catalysts.

[11] Yamada H. (2019). Improving Methodology of Particulate Measurement in Periodic Technical Inspection with High-Sensitivity Techniques: Laser Light Scattering Photometry and Particle Number Method. Emiss. Control Sci. Technol..

[12] Merkel G.A., Cutler W.A., Warren C.J. (2001). Thermal Durability of Wall-Flow Ceramic Diesel Particulate Filters. SAE Tech. Pap..

[13] Maier N., Nickel K.G., Engel C., Mattern A. (2010). Mechanisms and Orientation Dependence of the Corrosion of Single Crystal Cordierite by Model Diesel Particulate Ashes. J. Eur. Ceram. Soc..

[14] Yang K., Fox J.T., Hunsicker R. (2016). Characterizing Diesel Particulate Filter Failure during Commercial Fleet Use Due to Pinholes, Melting, Cracking, and Fouling. Emiss. Control Sci. Technol..

[15] UN ECE Tampering of Emissions Control Systems. https://unece.org/DAM/trans/doc/2019/wp29grpe/GRPE-78-04e.pdf.

[16] Boveroux F., Cassiers S., Buekenhoudt P., Chavatte L., De Meyer P., Jeanmart H., Verhelst S., Contino F. (2019). Feasibility Study of a New Test Procedure to Identify High Emitters of Particulate Matter during Periodical Technical Inspection. SAE Tech. Pap..

[17] Burtscher H., Lutz T., Mayer A. (2019). A New Periodic Technical Inspection for Particle Emissions of Vehicles. Emiss. Control Sci. Technol..

[18] Michael C., Victor V.M., Pierre B., Carsten G., Jelica P., Dario M., Robert L., Barouch G., Massimo C., Marcos O.G. (2020). Joint Research Centre 2019 Light-Duty Vehicles Emissions Testing, JRC122035.

[19] Posada F., Bandivadekar A. Global Overview of On-Board Diagnostics (OBD) Systems for Heavy-Duty Vehicles. https://theicct.org/sites/default/files/publications/ICCT_Overview_OBD-HDVs_20150209.pdf.

[20] Zhang S., Zhao P., He L., Yang Y., Liu B., He W., Cheng Y., Liu Y., Liu S., Hu Q. (2020). On-Board Monitoring (OBM) for Heavy-Duty Vehicle Emissions in China: Regulations, Early-Stage Evaluation and Policy Recommendations. Sci. Total Environ..

[21] Carslaw D., Rhys-Tyler G. (2013). New Insights from Comprehensive On-Road Measurements of NOx, NO2 and NH3 from Vehicle Emission Remote Sensing in London, UK. Atmos. Environ..

[22] Gruening C., Bonnel P., Clairotte M., Giechaskiel B., Valverdre V., Zardini A., Carriero M. (2019). Potential of Remote Sensing Devices (RSDs) to Screen Vehicle Emissions.

[23] Ropkins K., DeFries T.H., Pope F., Green D.C., Kemper J., Kishan S., Fuller G.W., Li H., Sidebottom J., Crilley L.R. (2017). Evaluation of EDAR Vehicle Emissions Remote Sensing Technology. Sci. Total Environ..

[24] Kadijk G., Elstgeest M., Ligterink N.E., Van der Mark P.J. (2017). Investigation into a Periodic Technical Inspection (PTI) Test Method to Check for Presence and Proper Functioning of Diesel Particulate Filters in Light-Duty Diesel Vehicles–Part 2.

[25] Giechaskiel B., Lähde T., Suarez-Bertoa R., Valverdre V., Clairotte M. (2020). Comparisons of Laboratory and On-Road Type-Approval Cycles with Idling Emissions. Implications for Periodical Technical Inspection (PTI) Sensors. Sensors.

[26] Giechaskiel B., Lähde T., Suarez-Bertoa R., Clairotte M., Grigoratos T., Zardini A., Perujo A., Martini G. (2018). Particle Number Measurements in the European Legislation and Future JRC Activities. Combust. Engines.

[27] Giechaskiel B., Valverdre V., Kontses A., Melas A., Martini G., Balazs A., Andersson J., Samaras Z., Dilara P. (2021). Particle Number Emissions of a Euro 6d-Temp Gasoline Vehicle under Extreme Temperatures and Driving Conditions. Catalysts.

[28] Mayer A., Lutz T., Spielvogel J., Franken O., Heuser M. (2021). PTI by Particle Count PN at Low Idle.

[29] Kupper M., Kraft M., Boies A., Bergmann A. (2020). High-Temperature Condensation Particle Counter Using a Systematically Selected Dedicated Working Fluid for Automotive Applications. Aerosol Sci. Technol..

[30] Giechaskiel B., Wang X., Horn H.G., Spielvogel J., Gerhart C., Southgate J., Jing L., Kasper M., Drossinos Y., Krasebrink A. (2009). Calibration of Condensation Particle Counters for Legislated Vehicle Number Emission Measurements. Aerosol Sci. Technol..

[31] Fierz M., Meier D., Steigmeier P., Burtscher H. (2014). Aerosol Measurement by Induced Currents. Aerosol Sci. Technol..

[32] Schriefl M.A., Bergmann A. Design Principles for Diffusion Chargers Sensing Particle Number Concentration. Proceedings of the 2016 IEEE Sensors.

[33] Rüggeberg T., Fierz M., Burtscher H., Melas A.D., Deloglou D., Papaioannou E., Konstandopoulos A.G. Application of DC-Sensors to Measure Ultrafine Particles from Combustion Engines. Proceedings of the 23rd Transport and Air Pollution Conference.

[34] Giechaskiel B., Drossinos Y. (2010). Theoretical Investigation of Volatile Removal Efficiency of Particle Number Measurement Systems. SAE Int. J. Engines.

[35] Burtscher H., Baltensperger U., Bukowiecki N., Cohn P., Hüglin C., Mohr M., Matter U., Nyeki S., Schmatloch V., Streit N. (2001). Separation of Volatile and Non-Volatile Aerosol Fractions by Thermodesorption: Instrumental Development and Applications. J. Aerosol Sci..

[36] Khalek I.S., Kittelson D.B. Real Time Measurement of Volatile and Solid Exhaust Particles Using a Catalytic Stripper. Proceedings of the International Congress & Exposition.

[37] Amanatidis S., Ntziachristos L., Giechaskiel B., Katsaounis D., Samaras Z., Bergmann A. (2013). Evaluation of an Oxidation Catalyst (‘Catalytic Stripper’) in Eliminating Volatile Material from Combustion Aerosol. J. Aerosol Sci..

[38] Melas A.D., Koidi V., Deloglou D., Daskalos E., Zarvalis D., Papaioannou E., Konstandopoulos A.G. (2020). Development and Evaluation of a Catalytic Stripper for the Measurement of Solid Ultrafine Particle Emissions from Internal Combustion Engines. Aerosol Sci. Technol..

[39] Giechaskiel B., Melas A.D., Lähde T., Martini G. (2020). Non-Volatile Particle Number Emission Measurements with Catalytic Strippers: A Review. Vehicles.

[40] Giechaskiel B., Lähde T., Melas A.D., Valverdre V., Clairotte M. (2021). Uncertainty of Laboratory and Portable Solid Particle Number Systems for Regulatory Measurements of Vehicle Emissions. Environ. Res..

[41] Yamada H., Funato K., Sakurai H. (2015). Application of the PMP Methodology to the Measurement of Sub-23 Nm Solid Particles: Calibration Procedures, Experimental Uncertainties, and DATA Correction Methods. J. Aerosol Sci..

[42] Giechaskiel B., Mamakos A., Andersson J., Dilara P., Martini G., Schindler W., Bergmann A. (2012). Measurement of Automotive Nonvolatile Particle Number Emissions within the European Legislative Framework: A Review. Aerosol Sci. Technol..

[43] Giechaskiel B., Lähde T., Schwelberger M., Kleinbach T., Roske H., Teti E., van den Bos T., Neils P., Delacroix C., Jakobsson T. (2019). Particle Number Measurements Directly from the Tailpipe for Type Approval of Heavy-Duty Engines. Appl. Sci..

[44] Terres A., Giechaskiel B., Nowak A., Ebert V. (2021). Calibration Uncertainty of 23nm Engine Exhaust Condensation Particle Counters with Soot Generators: A European Automotive Laboratory Comparison. Emiss. Control Sci. Technol..

[45] Amanatidis S., Maricq M.M., Ntziachristos L., Samaras Z. (2017). Application of the Dual Pegasor Particle Sensor to Real-Time Measurement of Motor Vehicle Exhaust PM. J. Aerosol Sci..

[46] Wang J., McNeill V.F., Collins D.R., Flagan R.C. (2002). Fast Mixing Condensation Nucleus Counter: Application to Rapid Scanning Differential Mobility Analyzer Measurements. Aerosol Sci. Technol..

[47] Bainschab M., Schriefl M.A., Bergmann A. (2020). Particle Number Measurements within Periodic Technical Inspections: A First Quantitative Assessment of the Influence of Size Distributions and the Fleet Emission Reduction. Atmos. Environ..

[48] Jarosinski W., Wisniowski P. (2021). Verifying the Efficiency of a Diesel Particulate Filter Using Particle Counters with Two Different Measurements in Periodic Technical Inspection of Vehicles. Energies.

[49] Suarez-Bertoa R., Lähde T., Giechaskiel B. Verification of NPTI-Instruments for Diesel and Petrol Vehicles—First Results. Presented at the ETH Conference on Nanoparticles.

[50] Schriefl M.A., Bergmann A., Fierz M. (2019). Design Principles for Sensing Particle Number Concentration and Mean Particle Size with Unipolar Diffusion Charging. IEEE Sens. J..

[51] Burtscher H. (2005). Physical Characterization of Particulate Emissions from Diesel Engines: A Review. J. Aerosol Sci..

[52] Amanatidis S., Ntziachristos L., Giechaskiel B., Bergmann A., Samaras Z. (2014). Impact of Selective Catalytic Reduction on Exhaust Particle Formation over Excess Ammonia Events. Environ. Sci. Technol..

[53] Mamakos A., Schwelberger M., Fierz M., Giechaskiel B. (2019). Effect of Selective Catalytic Reduction on Exhaust Nonvolatile Particle Emissions of Euro VI Heavy-Duty Compression Ignition Vehicles. Aerosol Sci. Technol..

[54] De Filippo A., Maricq M.M. (2008). Diesel Nucleation Mode Particles: Semivolatile or Solid?. Environ. Sci. Technol..

[55] Giechaskiel B., Lähde T., Drossinos Y. (2019). Regulating Particle Number Measurements from the Tailpipe of Light-Duty Vehicles: The next Step?. Environ. Res..

